# LAG-3 Blockade with Relatlimab (BMS-986016) Restores Anti-Leukemic Responses in Chronic Lymphocytic Leukemia

**DOI:** 10.3390/cancers13092112

**Published:** 2021-04-27

**Authors:** Christian Sordo-Bahamonde, Seila Lorenzo-Herrero, Ana P. González-Rodríguez, Ángel R. Payer, Esther González-García, Alejandro López-Soto, Segundo Gonzalez

**Affiliations:** 1 Department of Functional Biology, Immunology, Universidad de Oviedo, 33006 Oviedo, Spain; Christiansbl87@gmail.com (C.S.-B.); seilalorenzoherrero@gmail.com (S.L.-H.); 2 Instituto Universitario de Oncología del Principado de Asturias (IUOPA), 33006 Oviedo, Spain; apayer.angel@gmail.com (Á.R.P.); lopezsalejandro@uniovi.es (A.L.-S.); 3 Instituto de Investigación Sanitaria del Principado de Asturias (ISPA), 33011 Oviedo, Spain; 4 Department of Hematology, Hospital Universitario Central de Asturias (HUCA), 33011 Oviedo, Spain; 5 Department of Hematology, Hospital de Cabueñes, 33203 Gijón, Spain; esthergongar@yahoo.es; 6 Department of Biochemistry and Molecular Biology, University of Oviedo, 33006 Oviedo, Spain

**Keywords:** chronic lymphocytic leukemia, CLL, LAG3, relatlimab, immunotherapy, immune checkpoint, ICB, NK cell, immunosurveillance

## Abstract

**Simple Summary:**

Patients with chronic lymphocytic leukemia (CLL), the most frequent B cell malignancy in western countries, develop a progressive immunosuppression, leading to diminished anti-tumor immunity. Within the last years, immune checkpoint blockade has revolutionized anti-cancer therapies. Nonetheless, patients with CLL failed to achieve clinical benefits from therapies targeting widely-studied checkpoints such as PD-1/PD-L1 or CTLA-4. In this context, our results provide new insights about LAG-3 expression dysregulation in CLL and its role promoting tumor escape. Our data suggest that increased LAG-3 expression on leukemic cells correlates with shorter time to treatment and poor outcome in CLL. Moreover, treatment with relatlimab, a novel anti-LAG-3 blocking monoclonal antibody currently under clinical trial for different solid and hematological malignancies including CLL, restored, at least in part, NK and T cell-mediated anti-tumor responses. Altogether, our data provide the rationale to further investigate the role of LAG-3 in the pathogenesis of CLL.

**Abstract:**

The inclusion of monoclonal antibodies targeting immune checkpoints such PD-1/PD-L1 or CTLA-4 has revolutionized the landscape of anti-cancer therapy. However, PD-1 and CTLA-4 blockade failed to achieve clinical benefit in CLL, thus attention has been focused on emerging checkpoints in this malignancy. LAG-3 is an immune checkpoint receptor that negatively regulates T cell-mediated responses by inducing an hyporesponsive state, thus promoting tumor escape. Patients with chronic lymphocytic leukemia (CLL) develop a profound immune suppression that leads to lessened immunosurveillance and increased risk of developing a secondary neoplasia. In the study herein, we report the profound dysregulation of LAG-3 on leukemic cells in CLL. Likewise, natural killer (NK) and T cells showed increased LAG-3 expression, hence suggesting a role for this checkpoint in CLL-associated immunosuppression. High LAG-3 expression, as well as high levels of soluble LAG-3 (sLAG-3), correlated with adverse cytogenetics and poor outcome in patients with CLL, highlighting the clinical relevance of this immune checkpoint. Treatment of peripheral blood mononuclear cells (PBMCs) from patients with CLL with relatlimab, a new anti-LAG-3 blocking antibody currently evaluated in numerous clinical trials, depleted leukemic cells and restored NK cell- and T cell-mediated responses. Moreover, combination of LAG-3 with the immunomodulatory drug (IMiD) lenalidomide significantly increased IL-2 production by T cells and antibody-dependent cytotoxicity (ADCC) mediated by NK cells. Altogether, these data provide new insights into the potential anti-leukemic effects of relatlimab, currently in clinical trials in CLL, and provides the rationale to further investigate its combination with IMiDs for the management of hematological malignancies.

## 1. Introduction

Chronic lymphocytic leukemia (CLL), the most common B cell malignancy in adulthood, is characterized by a progressive accumulation of clonal mature B cells in blood, lymph nodes and bone marrow. T and natural killer (NK) cells have been shown to orchestrate antileukemic immune responses, hence playing a significant role in the pathogenesis of CLL [1,2,3]. However, CLL progression is associated with progressive immunosuppression, leading to attenuated immunosurveillance and diminished antitumor responses associated with an exhausted phenotype or unresponsive state of T and NK cells [4,5]. Thus, T and NK cells in CLL typically exhibit upregulation of inhibitory checkpoints, impaired proliferative capacity, reduced cytokine production and weak cytotoxicity against tumor cells [6,7,8,9]. Restoring T and NK cell-mediated responses through checkpoint blockade has become a promising therapeutic strategy in a wide variety of solid tumors and hematological malignancies [10]. However, immune checkpoint blockade (ICB)-based therapy using monoclonal antibodies (mAbs) against PD-1 and CTLA-4 failed to achieve clinical benefit in CLL [11,12]. In this context, exploring emerging checkpoint molecules as potential novel therapeutic targets in CLL has attracted attention in the last years.

Lymphocyte activation gene 3 (LAG-3) is an inhibitory immune checkpoint receptor that belongs to the immunoglobulin superfamily with approximately 20% amino acid homology with CD4. LAG-3 is expressed on activated and exhausted T and NK cells, as well as on B cells, dendritic cells, and regulatory T (Treg) cells [13,14,15,16]. Upon engagement with MHC class II molecules, or its newly described ligand, fibrinogen-like protein 1 (FGL1), LAG-3 negatively regulates T and NK cells, thereby promoting tumor escape likely by promoting exhaustion in combination with other checkpoints such as PD1 [17,18,19]. Hence, dysregulated LAG-3 expression has been reported to negatively correlate with clinical outcome in a wide variety of cancers, including some of hematological origin, including CLL, follicular lymphoma (FL), diffuse large B-cell lymphoma (DLBCL) or acute myeloid leukemia (AML) [20,21,22]. LAG-3 signaling impairs T cell proliferation, cytokine production and cytolytic function while its expression on Tregs may promote immunosuppression [23,24,25,26,27,28]. Despite the functional consequence of LAG-3 blockade has extensively been studied in T cells, it has poorly been explored in NK cell function, although LAG-3 blockade failed to increase NK cell-mediated cytotoxicity in previous studies [29,30,31].

Relatlimab (BMS-986016, Bristol-Myers Squibb, New York, NY, USA), a human IgG_4_ anti-LAG3 blocking mAb is currently being evaluated in several phase I and II/III clinical trials in solid tumors and hematological malignancies, including CLL (ClinicalTrials.gov Identifier: NCT02061761), alone or in combination with anti-PD-1/PD-L1 blocking mAbs [32].

Herein, we provide new insights into the potential role of relatlimab in the restoration of T and NK cell-mediated responses in patients with CLL. Our data confirm the profound dysregulation of LAG-3 in leukemic cells, and also unveil a significant dysregulation of LAG-3 on NK and T cells. Importantly, this dysregulation correlates with adverse genetic and cytogenetic features of patients with CLL and it is associated with poor outcome. LAG-3 inhibitory signaling disruption with relatlimab significantly enhanced antitumor responses mediated by NK and T cells in CLL, further supporting the use of anti-LAG-3 mAbs in the treatment of CLL.

## 2. Materials and Methods

### 2.1. Patient’s Samples

Peripheral blood samples from 61 consecutive non-treated patients with CLL (Table 1) were obtained from Hospital Universitario Central de Asturias and Hospital de Cabueñes according to the Declaration of Helsinki with the approval of the local ethics committee (Comité de Ética de la Investigación del Principado de Asturias, case-19042016). Clinical analyses were performed according to International workshop on CLL guidelines criteria. Samples from healthy donors (HD) (*n* = 20) were provided by Centro Comunitario de Sangre y Tejidos de Asturias. FISH analysis for chromosome 13q deletion, 11q deletion, 17p deletion and trisomy 12 was performed. Variable region of the immunoglobulin heavy chain *IGHV* mutation status was characterized by direct sequencing method, and patients were categorized as unmutated (IGHV 98% germline homology) or mutated (98% homology). Twenty patients showed clinical progression and required therapeutic intervention after sample collection. The median follow-up from diagnosis of patients was 71 months.

Peripheral blood mononuclear cells (PBMCs) from patients with CLL and HD were isolated by ficoll density gradient centrifugation ((Histopaque^®^-1077, Sigma-Aldrich, St. Louis, MO, USA) and used fresh. PBMCs were cultured in RPMI 1640 (Lonza, Basilea, Switzerland) complete medium (supplemented with 10% heat-inactivated fetal bovine serum, 1 mM sodium pyruvate, 2 mM L-glutamine, 100 U/mL penicillin and 10 μg/mL streptomycin) at 37 °C and 5% CO_2_. CLL-derived cell line MEC-1 was obtained from the ATCC and cultured in Iscove’s Modified Dulbecco’s Medium (IMDM) supplemented as described above.

### 2.2. Immune Subset Identification and Phenotypical Analyses

Phenotypical analyses of leukemic cells, B lymphocytes and T and NK cells from patients and HD were performed by flow cytometry. The following antibodies were employed: anti-CD19-APC, anti-CD4-CFblue, anti-CD8-APC750 and anti-CD3-PE (Immunostep, Salamanca, Spain), anti-CD3-FITC and anti-CD56-APC (both from Cytognos, Salamanca, Spain). Leukemic cells were identified as CD19+. NK cells were defined as CD56+CD3−. Total T lymphocytes (CD3+CD56−) as well as T helper (CD3+CD4+) and cytotoxic (CD3+CD8+) subpopulations were also identified. LAG-3 expression was evaluated using anti-LAG3-PE (clone 7H2C65, Biolegend, San Diego, CA, USA). Data was analyzed in a Cytoflex S flow cytometer and CytExpert 2.3 software (Beckman Coulter, Brea, CA, USA). 

### 2.3. Absolute Cell Count Assay

PBMCs from patients with CLL were treated with the blocking anti-LAG-3 mAb relatlimab (BMS-986016, kindly provided by Bristol-Myers Squibb), or control IgG (10 µg/mL) for 72 h or a week, alone or in combination with 10 µM lenalidomide (LND) (Santa Cruz Biotechnology, Dallas, TX, USA). At the indicated timepoint, leukemic cells were identified with anti-CD19-APC staining and an equal volume of PKH26 reference microbeads (Sigma-Aldrich) was added to each condition. A total of 5000 reference microbeads were acquired, and absolute leukemic cell count was determined by flow cytometry. Percentage of leukemic cell depletion was normalized to control.

### 2.4. Proliferation Assay

PBMCs obtained from patients were stained with 1 µM 5,6-carboxyfluorescein diacetate succinimidyl ester (CFSE, Sigma Aldrich) and cultured in the presence or absence of anti-LAG-3 blocking antibody or control IgG for 72 h. Culture media was supplemented with 50 U/mL IL-2 (ORF Genetics, Kópavogu, Iceland). Immune subsets were identified as mentioned above and percentage of proliferating cells was analyzed by flow cytometry.

### 2.5. In Silico Analysis 

*LAG3* mRNA expression was evaluated from publicly available RNAseq data from the Gene Expression Omnibus repository (GEO). GSE22762, GSE4392, GSE112953 and GSE1590 datasets were analyzed using the online webtool ShinyGeo (https://gdancik.shinyapps.io/shinyGEO/, accessed on 8 January 2020) [33]. The prognostic value of *LAG3* axis was evaluated using Prediction of Clinical Outcomes from Genomic Profiles (PRECOG) tool. *Z*-scores for each gene studied were obtained from the PRECOG website and a heatmap was drawn using GraphPad Prism 8.0 software (Biosoft, San Diego, CA, USA) [34]. The mutational landscape regarding *LAG3* expression was evaluated using cBioportal v3.6.12 (https://www.cbioportal.org/ accessed on 12 December 2019) publicly available data [35].

### 2.6. Evaluation of Soluble LAG-3 in Serum Samples

Levels of soluble LAG-3 (sLAG-3) were evaluated in serum samples from 28 patients with CLL and 12 HD by ELISA (RayBiotech, Peachtree Corners, GA, USA) following the manufacturer’s instructions.

### 2.7. Intracellular Protein Staining

To assess the effect of relatlimab on cytokine production, PBMCs from patients with CLL were treated with anti-LAG-3 blocking mAb or control IgG (10 µg/mL) alone or in combination with lenalidomide (10 µM) or DMSO for 72 h. Then, immune subset identification followed by intracellular cytokine staining and flow cytometry analyses were performed as previously described by our group [36]. Briefly, treated PBMCs were stimulated with 50 nM PMA and 1 µg/mL ionomycin for 4 h. After 1 h incubation with PMA/ionomycin, brefeldin A (Biolegend) was added. Afterwards, BD Cytofix/Cytoperm Fixation/Permeabilization Kit (BD Biosciences, BD Biosciences, San Jose, CA, USA) was employed according to the manufacturer’s instructions. Percentage of positive T lymphocytes for IL-2 (clone MQ1-17H12, Biolegend), IFN-γ (clone 4S.B3, Biolegend) and TNF-α (clone Mab11, Biolegend) staining was determined. For evaluation of intracellular Bcl-2 protein levels, PBMCs from patients were treated with relatlimab or control IgG (10 µg/mL) for 72 h or 7 days. Thereupon, cells were stained with anti-CD19-APC for leukemic cell identification and BD Cytofix/Cytoperm Kit was used to fix and permeabilize cells. Bcl-2 expression was evaluated using anti-Bcl-2-PE (clone 100, Biolegend).

### 2.8. Determination of NK Cell-Mediated Cytotoxicity

NK cell-mediated cytotoxicity and antibody-dependent cellular cytotoxicity (ADCC) was evaluated by calcein-AM staining as previously described by our group [37]. Total PBMCs from patients with CLL were treated with relatlimab or control IgG (10 μg/mL) for 72 h. MEC-1 target cell line was stained with 10 µM calcein-AM (Biolegend) according to manufacturer’s instructions and co-cultured with PBMCs at a 25:1 effector: target (E:T) ratio for 4 h in a 96-well U-bottom plate, and calcein release was measured on a Varioskan^™^ LUX multimode microplate reader. For ADCC assays, MEC-1 cell line was pre-treated with 10 μg/mL rituximab or control IgG for 30 min.

### 2.9. Statistics 

Statistical analyses were performed using GraphPad Prism 8.0 software (Biosoft). The relationship between continuous and categorical prognostic variables was evaluated by Mann–Whitney *U*-test, whereas intra-group comparisons were performed by Wilcoxon Matched-Pairs Signed Ranks test. For time-to-event analysis, Kaplan–Meier curves were plotted, and each group was compared by long-rank test. Relationship between patient characteristics and survival was evaluated by univariate Cox proportional hazards models using SPSS v23.0 software; *p*-values of ≤0.05 were considered statistically significant.

## 3. Results

### 3.1. Surface and Soluble LAG-3 Expression Are Increased in CLL 

**Figure 1:**
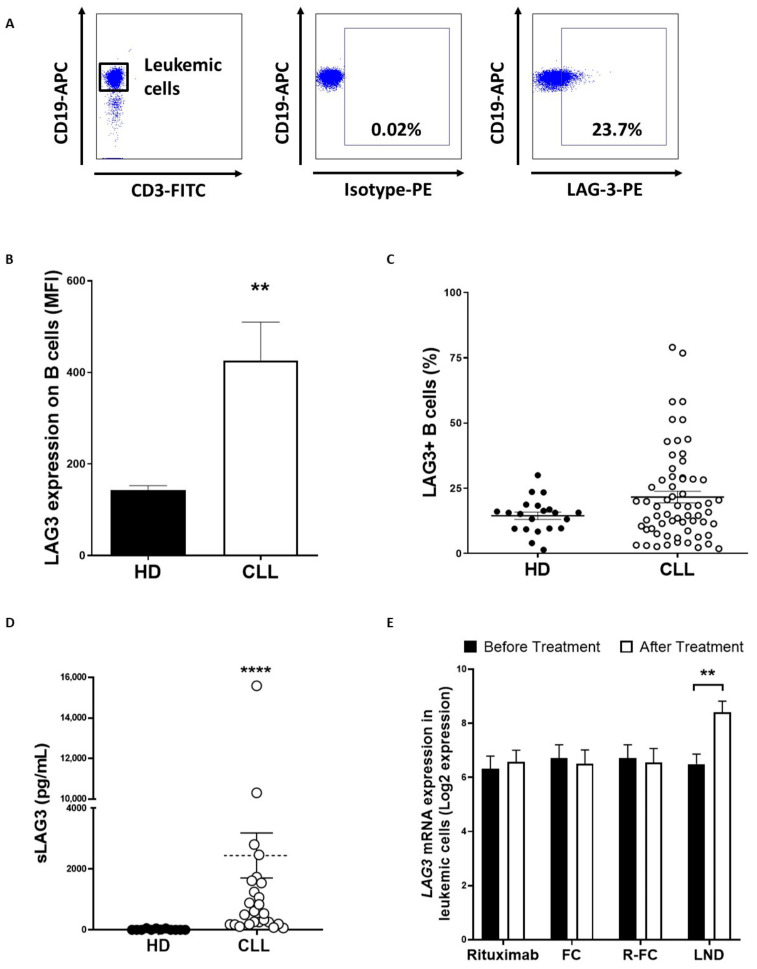
LAG-3 expression is upregulated on leukemic cells from patients with CLL. LAG-3 surface expression was evaluated on PBMCs from patients with CLL (*n* = 61) and HD (*n* = 20) by flow cytometry. (**A**) Representative dot plot for LAG-3+ CD19+ analysis; (**B**) Comparison of LAG-3 expression (MFI ± SEM) between leukemic cells and B lymphocytes from HD; (**C**) Percentage of LAG-3+ leukemic cells compared to B cells from HD; (**D**) Sera levels of sLAG-3 were evaluated in 28 patients with CLL and 12 HD; (**E**) In silico analysis of the effect of the indicated therapies on *LAG3* mRNA expression in leukemic cells. GSE112953 and GSE58211 from GEO database were included. Each dot represents an individual sample (FC: fludarabine-chlorambucil, R-FC: Rituximab- fludarabine-chlorambucil, LND: lenalidomide). ** *p* < 0.01 and **** *p* < 0.0001.

LAG-3 surface expression was evaluated in PBMCs from patients with CLL (*n* = 61) and HD (*n* = 20) by flow cytometry (Figure 1A). While no significant differences in the percentage of LAG-3 expressing B cells were observed, the level of LAG3 surface expression was found to be significantly upregulated on leukemic cells from patients with CLL (mean fluorescence intensity (MFI) ± standard error of mean (SEM): 426.4 ± 83.5 vs. 142.7 ± 10.1, *p* = 0.0012) (Figure 1B,C). In line with this, sLAG3 levels were highly increased in serum samples from patients compared to HD (2445 ng/mL ± 741 ng/mL vs. 13.05 ng/mL ± 6.3 ng/mL, *p* < 0.0001) (Figure 1D). Remarkably, levels of sLAG-3 positively correlated with surface expression and percentage of LAG-3-+ leukemic cells (Spearman’s rank test *p* = 0.002 and *p* = 0.006, respectively). In silico analysis of publicly available data from GEO database revealed that treatment of patients with lenalidomide, but not rituximab or fludarabine-chlorambucil regimen, significantly increased *LAG3* mRNA expression in leukemic cells (*p* = 0.0024) (Figure 1E). Altogether these data confirm the profound dysregulation of LAG-3 on leukemic cells and provides the rationale for the investigation of LAG-3 blockade in combination with the immunomodulatory drug lenalidomide [22]. 

### 3.2. LAG-3 Expression Correlates with Adverse Clinical Features and Poor Outcome in CLL

**Figure 2:**
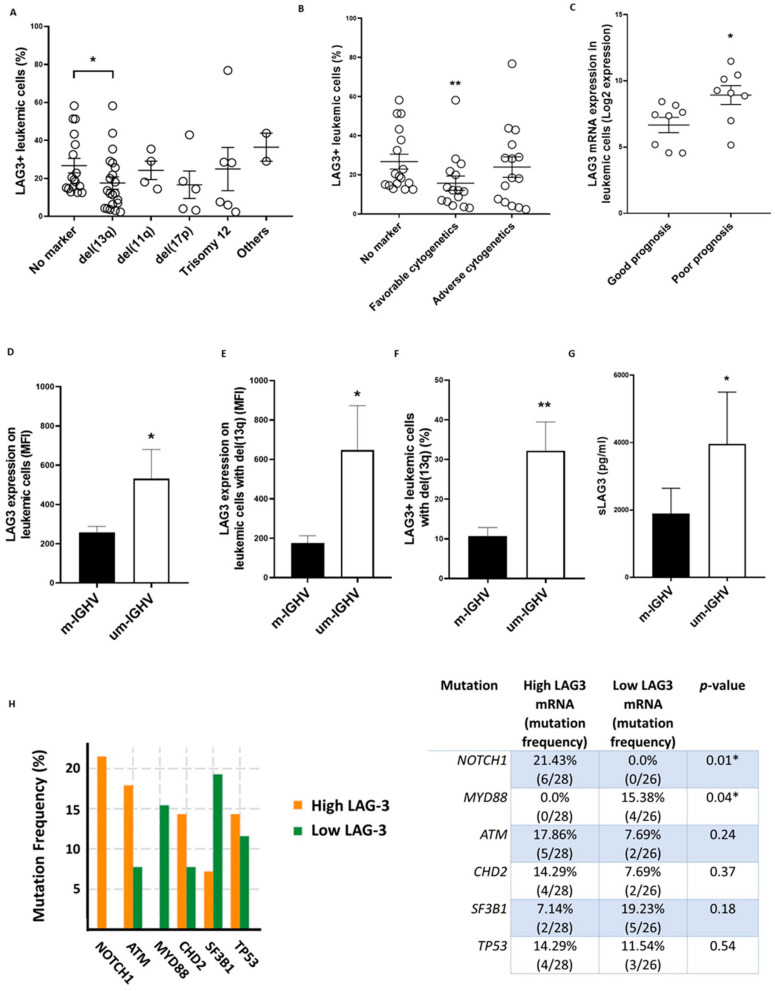
LAG-3 expression correlates with prognostic factors in CLL. (**A**) LAG-3 surface expression was evaluated on leukemic cells from patients with CLL (*n* = 61) categorized by cytogenetics abnormalities by flow cytometry; (**B**) LAG-3 surface expression on leukemic cells in patients stratified by cytogenetics abnormalities (*n* = 61): no marker (no abnormalities), favorable cytogenetics (del(13q)) and adverse cytogenetics (patients with del(11q), del(17p) and/or trisomy 12 owing or not del(13q)) is shown; (**C**) In silico analysis (GSE4392) of *LAG3* mRNA expression in leukemic cells from ZAP70-CD38- (good prognosis) and ZAP70+CD38+ (poor prognosis) patients; (**D**–**G**) Evaluation of LAG-3 expression on leukemic cells from patients with mutated (m-IGHV) or unmutated (um-IGHV) *IGHV*; Comparison of LAG-3 expression patients (MFI ± SEM) (**E**) and percentage (**F**) in patients with del(13q) are shown (*n* = 61); (**G**) Sera levels of sLAG-3 in 28 patients with mutated and unmutated *IGHV* status are depicted; (**H**) In silico analysis of *LAG3* mRNA expression in patients stratified by driver mutations in CLL using cBioportal. * *p* < 0.05 and ** *p* < 0.01.

Patients were next stratified by their cytogenetic alterations, revealing a diminished percentage of LAG-3+ leukemic cells in patients carrying del(13q) (17.5% ± 3.2 vs. 26.7% ± 3.8, *p* = 0.02) (Figure 2A). No significant differences were observed in those patients carrying adverse cytogenetics (patients with del(11q), del(17p) and/or trisomy 12 owing or not del(13q)) (Figure 2B) [38]. In silico analyses of RNAseq data from GEO database revealed that patients with ZAP70-CD38- (good prognosis) exhibited lower *LAG3* mRNA levels than those expressing ZAP70 + CD38 + (poor prognosis) (Figure 2C).

As previously reported, increased LAG-3 expression was detected on leukemic cells from patients with unmutated *IGHV* (Figure 2D) [22]. Further, patients with del(13q) and unmutated *IGHV* displayed augmented LAG-3 expression compared to patients with del(13q) and mutated status, suggesting that the levels of expression of this checkpoint mainly rely on mutational status rather than chromosomic alterations (Figure 2E,F). In addition, sLAG3 levels were also increased in patients with unmutated *IGHV* status (Figure 2G). Besides, in silico analyses using available data from the TCGA revealed that increased *LAG-3* expression in leukemic cells was more abundant in patients bearing unfavorable mutations affecting *NOTCH1* and *MYD88* genes (*p* = 0.01 and *p* = 0.04, respectively) (Figure 2H) [38]. Taken together, these findings suggest a higher dysregulation of LAG-3 expression on leukemic cells in patients with poor prognosis and unfavorable genetic and cytogenetic features.

### 3.3. High LAG-3 Expression Is Associated with Shorter Time to Treatment and Poorer Overall Survival in CLL

**Figure 3:**
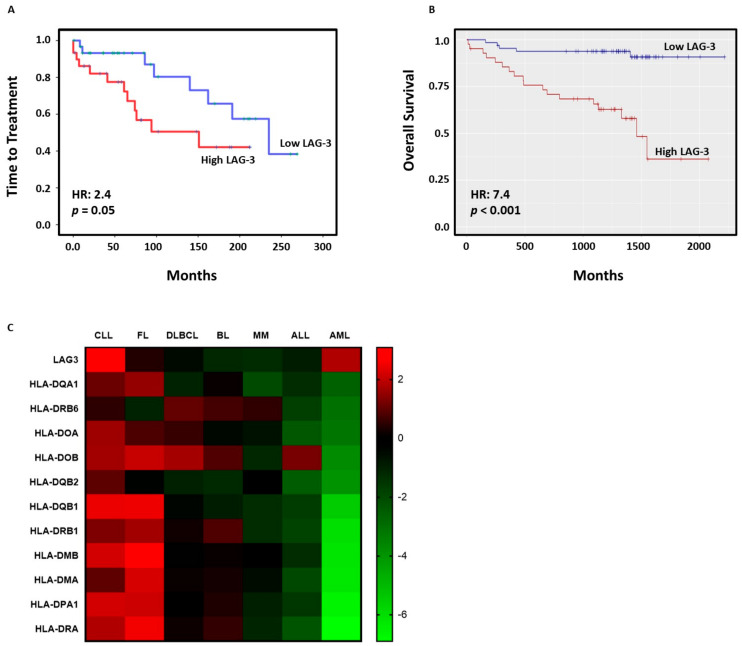
High LAG-3 expression on leukemic cells correlates with poor outcome in CLL. (**A**) Kaplan-Meier survival analysis relative to LAG-3 surface expression and TTT in 61 patients with CLL; (**B**) In silico analysis of OS in 107 patients with CLL using ShinyGeo online tool (GSE22762); (**C**) Heatmap representing z-score corresponding to the expression of *LAG3* and MHC class II genes and its influence in OS in diverse hematological malignancies analyzed by PRECOG tool (CLL: chronic lymphocytic leukemia; FL: follicular lymphoma; DLBCL: diffuse large B-cell lymphoma; BL: Burkitt leukemia/lymphoma; MM: multiple myeloma; ALL: acute lymphoblastic leukemia; AML: acute myeloid leukemia).

The impact of LAG-3 expression on survival was next evaluated. Noticeably, high LAG-3 surface expression on leukemic cells was associated with shorter time to treatment (TTT) (hazard risk (HR) = 2.4, *p* = 0.05) in our cohort (Figure 3A). In agreement, survival analysis using *LAG3* mRNA expression from 107 patients with CLL (GSE22762 dataset) revealed diminished overall survival (OS) in patients with high *LAG3* expression (HR = 7.4, *p* < 0.001) (Figure 3B). The influence of *LAG3* and MHC class II genes on outcome in different hematological malignancies was further analyzed using the PRECOG tool. Z-score analysis, which allows direct comparison across different studies and platforms independently from timescale and range of predictor variables, revealed that *LAG3* axis has more influence on the clinical outcome of patients with CLL than in other hematological malignancies (Figure 3C). Altogether, these data highlight the importance of LAG-3 signaling in CLL and provides the rationale to further investigate this checkpoint as a possible therapeutic target in CLL. 

### 3.4. LAG-3 Expression Is Dysregulated on T and NK Cells and Affects Their Proliferation

**Figure 4:**
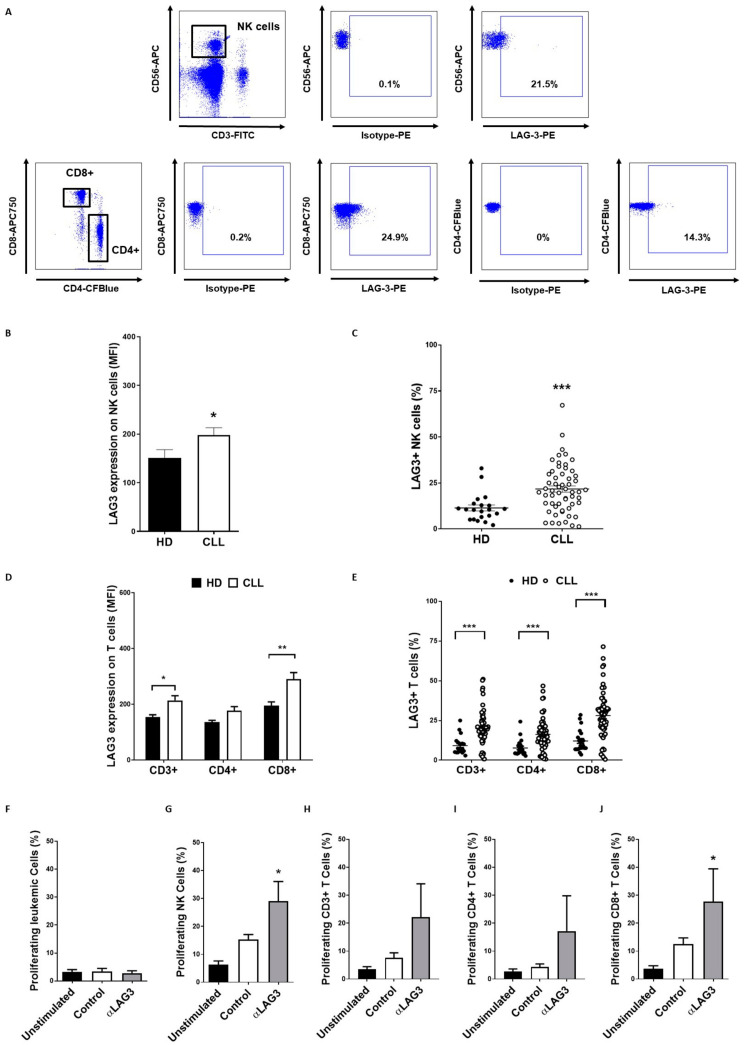
LAG-3 expression is increased on NK and T cells and induces immunosuppression. (**A**) Representative dot plots of percentage of LAG-3+ NK and CD8+ T cells in PBMCs from patients with CLL; LAG-3 surface expression was evaluated in 59 patients and 20 healthy donors in NK (**B**,**C**) and T cells (**D**,**E**) by flow cytometry. The graphs represent MFI ± SEM and percentage of LAG-3+ cells; The effect of LAG-3 blockade with relatlimab or isotype control (Control; 10 µg/mL) on the proliferation of leukemic cells (**F**), NK cells (**G**) or T cells (**H**–**J**) stimulated with 50 U/mL IL-2 was evaluated by CFSE staining and flow cytometry (*n* = 6). * *p* < 0.05, ** *p* < 0.01 and *** *p* < 0.001.

Surface LAG-3 expression on T and NK cells was next evaluated in 59 non-treated patients with CLL and 20 HD by flow cytometry. LAG-3 surface expression on NK cells as well as percentage of LAG-3+ NK cells (21.7% ± 1.7 vs. 11.4% ± 1.6, *p* = 0.0004) were significantly increased in patients (Figure 4A–C). LAG-3 levels were also elevated in total CD3+ T lymphocytes and CD8+ effector T cells, but it did not reach statistical significance in CD4+ T cells (MFI: 213.8 ± 16.7 vs. 151.3 ± 8.3, *p* = 0.02, 290.1 ± 23.5 vs. 188.7 ± 14.2, *p* = 0.005 and 176.7 ± 17.78 vs. 132.9 ± 6.3, *p* = ns, respectively) (Figure 4A,D). However, patients with CLL exhibited heightened percentage of both LAG-3+ CD4+ T cells (16.3 ± 1.3 vs. 7.5 ± 1.1, *p* = 0.0001) and CD8+ T cells (28.2 ± 2.0 vs. 11.9 ± 1.5, *p* < 0.0001) (Figure 4E). Interestingly, percentage of LAG-3+ NK cells and CD4+ T cells positively correlated with LAG-3+ leukemic cells (Spearman’s rank test *p* = 0.01 and *p* = 0.03, respectively). Likewise, percentage of LAG-3+ NK cells highly correlated with LAG-3+ CD4+ (*r* = 0.8, *p* < 0.0001) and CD8+ T cells (*r* = 0.75, *p* < 0.0001), thus suggesting a common mechanism regulating LAG-3 expression on these immune subsets.

In order to evaluate the effect of increased LAG-3 expression on the above-mentioned immune subsets, PBMCs from 7 patients were treated with relatlimab (or control IgG) and stimulated with IL-2 for 72 h. Relatlimab significantly boosted the proliferation of NK cells (29.0 ± 7 vs. 15.3 ± 1.7, *p* = 0.03) and CD8+ effector T cells (27.69 ± 11.7 vs. 12.5 ± 2.2, *p* = 0.04) without affecting leukemic cell proliferation (2.7 ± 0.8 vs. 3.3 ± 1.1) (Figure 4F–J). These data suggest that LAG-3 exerts a co-inhibitory activity in NK cells and T cells from patients with CLL that may be reverted by relatlimab treatment.

### 3.5. Relatlimab Induces Leukemic Cell Depletion and Enhances ADCC 

**Figure 5:**
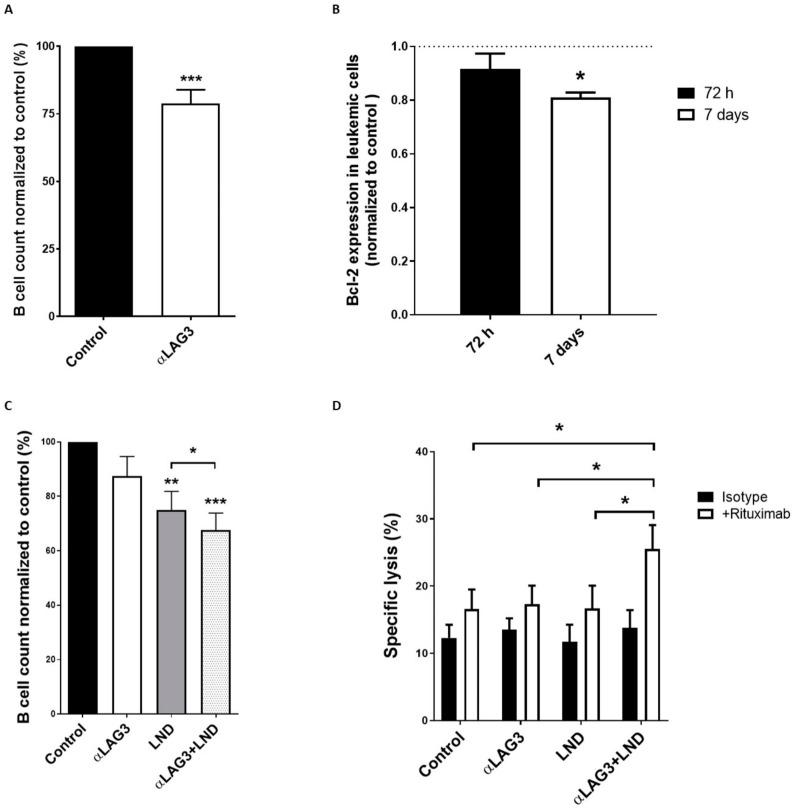
Treatment with relatlimab depletes leukemic cells. (**A**) PBMCs from 26 patients with CLL were treated with relatlimab or isotype control (10 µg/mL) and absolute leukemic cell count was performed at indicated timepoints. Bars represent the percentage of leukemic cells normalized to control; (**B**) Intracellular staining and flow cytometry analysis for Bcl-2 expression (normalized to control) were performed in PBMCs treated with relatlimab or isotype control (10 µg/mL) for 72 h or 7 days (*n* = 8); (**C**) Evaluation of absolute leukemic cell count (normalized to control) in PBMCs from patients treated with relatlimab or isotype control (10 µg/mL), alone or in combination with lenalidomide (LND) (10 µM), for 72 h (*n* = 12); (**D**) Effect of LAG-3 blockade on NK cell-mediated cytotoxicity was evaluated by calcein-AM assay in PBMCs from 10 patients treated with relatlimab (10 µg/mL), alone or in combination with lenalidomide (LND; 10 µM), and co-cultured with MEC-I CLL-derived cell line at 25:1 (E:T) ratio (*n* = 10) for 72 h. ADCC was evaluated by pre-treating target MEC-I cells with rituximab (10 µg/mL). * *p* < 0.05, ** *p* < 0.01 and *** *p* < 0.001.

In order to evaluate the effect of LAG-3 blockade on leukemic cell count, PBMCs obtained from 26 patients with CLL were treated with relatlimab for 7 days. The anti-LAG3 blocking mAb significantly reduced leukemic cell numbers compared to control IgG (Figure 5A). Interestingly, treatment with relatlimab reduced the expression of the anti-apoptotic protein Bcl-2, suggesting that LAG-3 blockade may impede pro-survival signaling in leukemic cells (Figure 5B). Further, combination of relatlimab with lenalidomide significantly increased the depletion of leukemic cells (Figure 5C). Despite no direct effect of LAG-3 blockade on NK cell-mediated cytotoxicity was observed, calcein-AM cytotoxicity assays revealed that combination of LAG-3 blockade and lenalidomide enhances rituximab-mediated ADCC activity of NK cells from patients with CLL (Figure 5D). Taken together, these data suggest that relatlimab, alone or in combination with lenalidomide, exhibit anti-leukemic activity that merits further investigations in CLL.

### 3.6. LAG-3 Blockade with Relatlimab Promotes Cytokine Production by T Cells

**Figure 6:**
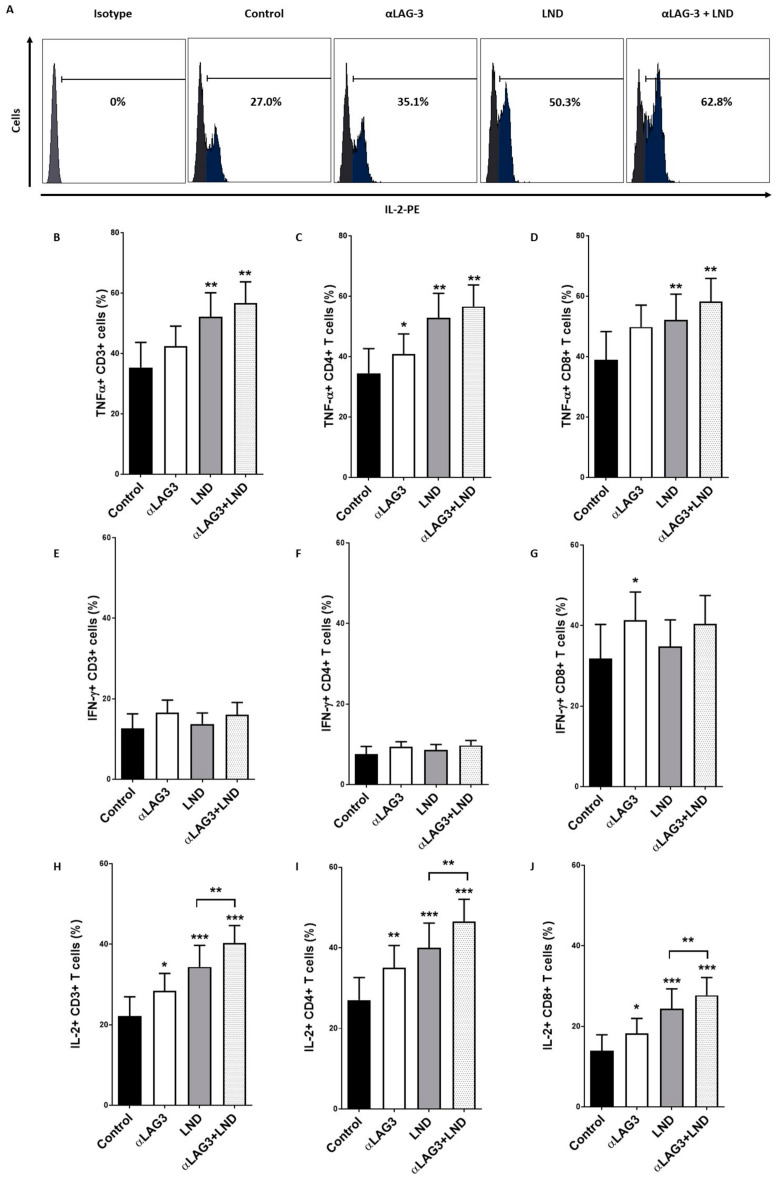
Relatlimab enhances cytokine production by T lymphocytes. (**A**) Representative histogram showing the effect of LAG-3 blockade, alone or in combination with lenalidomide, on IL-2 production by CD4+ T cells. (**B**–**J**) PBMCs from 11 patients with CLL were treated with relatlimab or isotype control (10 µg/mL), alone or in combination with lenalidomide (LND, 10 µM), for 72 h. The percentage of T cells expressing TNF-α (**B**–**D**), IFN-γ (**E**–**G**) and IL-2 (**H**–**J**) was evaluated by flow cytometry. * *p* < 0.05, ** *p* < 0.01 and *** *p* < 0.001.

PBMCs obtained from 11 patients with CLL were treated with relatlimab and T cell production of TNF-α, IFN-γ and IL-2 was measured by intracellular staining and flow cytometry analysis (Figure 6). LAG-3 blockade increased the percentage of TNF-α+ CD4+ T cells. Lenalidomide significantly boosted TNF-α production by both CD4+ and CD8+ T cells, but no cooperative effect with LAG-3 blockade was observed. LAG-3 blockade also increased the percentage of IFN-γ+ CD8+ T cells (41.3% ± 6.9 vs. 31.8% ± 8.4, *p* = 0.05), but no effect was observed upon lenalidomide treatment. Moreover, no effect on IFN-γ production by NK cells was observed. Importantly, treatment with relatlimab increased the production of IL-2 by T lymphocytes and, specifically, by CD4+/CD8+ T cell subsets (28.4% ± 4.3 vs. 22.1% ± 4.8, *p* = 0.003, 35.0% ± 5.5 vs. 27.0% ± 5.5, *p* = 0.002 and 18.2% ± 3.7 vs. 13.9% ± 3.9, *p* = 0.02, respectively). Furthermore, the combination of LAG-3 blockade and lenalidomide significantly strengthened the increase of IL-2+ T cells. Altogether, these data indicate that LAG-3 may have a co-inhibitory activity in the regulation of cytokine production by T cells in CLL and LAG-3 blockade with relatlimab may, at least partially, restore this crippled T cell-mediated immune response.

## 4. Discussion

Recent advances with small molecule inhibitors, such as ibrutinib or venetoclax, have changed the landscape of management of patients with CLL. However, the arising resistance mechanisms associated with these therapies, together with the profound immunosuppression related to this disease, encourage the need for exploring new therapeutic alternatives. ICB-based therapies have prompted a major breakthrough in the treatment of cancer. Nonetheless, no clinical benefits have been achieved with anti-PD-1/PD-L1 blocking mAbs in CLL. In this context, novel checkpoint molecules, including LAG-3, NKG2A or ILT2, have been proposed as potential therapeutic targets in CLL [22,39,40,41]. 

LAG-3 is, along with PD-1/PD-L1 and CTLA-4, a promising target in cancer immunotherapy, since it plays a pivotal role in anti-tumor immunity [42]. LAG-3 blockade, alone or in combination with other ICBs, has demonstrated promising results in clinical trials [17,19,42,43,44]. Yet, little is known about the mechanisms of action underlying LAG-3 blockade in cancer [43]. Relatlimab, an anti-LAG-3 blocking mAb, is currently one of the most advanced mAbs in clinical trials targeting solid tumors and hematological malignancies. Previous studies have reported the relevance of LAG-3/MHC-II in CLL pathology, highlighting this disease as a possible target for anti-LAG-3-based therapies. Our study confirms the profound dysregulation of LAG-3 expression on leukemic cells. Further, our data unveil that LAG-3 surface expression is decreased in patients with good prognostic features, such as those carrying del(13q) and mutated *IGHV*. Contrarily, LAG-3 expression is increased in patients with bad prognostic features, including unmutated *IGHV*, *NOTCH1* or *MYD88* mutations and ZAP70+CD38+ status. Accordingly, high LAG-3 surface expression correlated with shorter TTT in our cohort, and in silico analyses confirmed the poorer outcome in patients with high expression of LAG-3 and MHC-II genes in a larger set of patients with CLL. Importantly, heightened expression was also observed in T and NK cells, and LAG-3 expression on these immune subsets significantly correlated with LAG-3 expression on leukemic cells. Moreover, LAG-3 expression on NK cells highly correlated with LAG-3 expression on T cells, suggesting a common mechanism of regulation among leukemic and healthy immune cells. Altogether, these data highlight the dysregulation of LAG-3 on leukemic cells and provide new insights into the relevance of LAG-3 dysregulation in the clinical outcome of patients with CLL, hence suggesting that it may be involved in the suppression of the immune response in this malignancy. 

To further analyze the therapeutic potential of targeting LAG-3 in CLL, we analyzed the anti-leukemic effect of relatlimab, an anti-LAG-3 blocking antibody currently ongoing clinical trials in CLL (ClinicalTrials.gov Identifier: NCT02061761), as well as the potential underlying mechanisms leading to anti-tumor responses in patients with CLL. Foremost, we demonstrate that relatlimab induces the depletion of leukemic cells ex vivo. Multiple underlying mechanisms may account for this anti-leukemic effect. Firstly, LAG-3 blockade may block the proposed sLAG-3 autocrine loop that promotes anti-apoptotic signaling upon engagement with MHC-II, which agrees with the decreased Bcl-2 expression observed in leukemic cells [22]. Secondly, our data reveal that LAG-3 has an immunosuppressive role in CLL, since LAG-3 sharply inhibited T and NK cell proliferation and cytokine production by T cells. Consequently, relatlimab was able to restore T and NK cell-mediated anti-tumor responses. LAG-3 blockade sharply increased NK and CD8+ T cell proliferation, as well as promoted production of IL-2 and other cytokines by T cells. Although LAG-3 blockade, along with IL-12 treatment, was shown to restore the anti-metastatic activity of NK cells in murine models of breast cancer, relatlimab did not directly induce NK cell-mediated cytotoxicity against leukemic cells. This is in line with previous reports, suggesting that LAG-3 do not play a direct role enhancing NK cell-mediated cytotoxicity [29,30,45]. In silico analysis showed that lenalidomide, but not rituximab or fludarabine-chlorambucil regimen, significantly increased LAG-3 expression on leukemic cells, suggesting that LAG-3 blockade may potentiate the anti-tumor effect of the immunomodulatory drug. These data agree with prior works from our group and others bringing to light that IMiDs may strengthen ICB-induced responses and NK cell-mediated cytotoxicity in CLL [40,46,47]. Accordingly, combination of LAG-3 blockade and lenalidomide cooperatively depleted leukemic cells in vitro. Further, treatment with relatlimab, in combination with lenalidomide, resulted in stimulated IL-2 and IFN-γ production by T cells and increased antibody-dependent cytotoxicity (ADCC). This effect may be associated with enhanced anti-leukemic responses mediated by NK and T cells. Overall, our data suggest that LAG-3 blockade may have a co-stimulatory effect in NK cell cytotoxicity, and combination of LAG-3 blockade with immunomodulatory drugs or cytokine treatment may increase NK cell-mediated cytotoxicity. The elucidation of additional costimulatory drugs potentiating LAG-3 blockade, and the effect of combining LAG-3 blockade with immunomodulatory drugs in other hematological malignancies typically displaying LAG-3 dysregulation, such as multiple myeloma, warrants further investigations [48,49,50]. 

In sum, our study confirms the profound alteration of LAG-3 expression and its clinical and immune relevance in CLL. Further, we provide new insights on the anti-leukemic potential of relatlimab, which may be related to decreased anti-apoptotic signaling in malignant cells, as well as enhanced NK and T cell-mediated responses.

## Figures and Tables

**Table 1:** Clinical characteristics of patients with CLL included in the study.

Patients	Patients (*n* = 61)	%
Age		
Years (mean)	74.8	
Sex		
Female	24	39.3
Male	37	60.6
Rai Stage		
0	35	57.3
I–II	18	29.5
III–IV	8	13.1
Binet Stage		
A	49	80.3
B	6	9.8
C	6	9.8
Cytogenetic abnormalities (FISH)		
No alterations	26	42.6
del(13q)	18	29.5
del(11q)	1	1.6
del(17p)	3	4.9
Trisomy 12	5	8.1
Complex karyotype	8	13.1
*IGHV* status		
Mutated	37	60.6
Unmutated	13	21.3
Undetermined	11	18
Progression		
Stable disease	41	67.2
Progressive disease	20	32.7

*IGHV*: Variable region of the immunoglobulin heavy chain.

## Data Availability

In silico analysis were performed from publicly available datasets from Gene Expression Omnibus (GEO) repository using ShinyGeo (https://gdancik.shinyapps.io/shinyGEO/ accessed on 8 January 2020) and RNAseq data from the TCGA database using cBioportal (cbioportal.org accessed on 12 December 2019).
